# A Case of Recurrent Syncope Solely Associated With Air Travel

**DOI:** 10.1155/cric/3379568

**Published:** 2026-05-06

**Authors:** Varun Srivatsav, Brendon Macknak

**Affiliations:** ^1^ Division of Cardiology, Department of Medicine, Queen′s University, Kingston, Ontario, Canada; ^2^ Division of Cardiology, Department of Medicine, University of Saskatchewan, Regina, Saskatchewan, Canada, usask.ca

## Abstract

A 68‐year‐old patient was referred for recurrent episodes of syncope associated solely with air travel. He did not experience episodes of syncope in other situations and was well otherwise. His ECG, echocardiogram, exercise stress test and MIBI were unremarkable. It was determined that the patient had been experiencing repeated vasovagal syncope from an uncommon trigger of air travel. He responded to nonpharmacological measures to treat vasovagal syncope and doses of fludrocortisone the night before air travel, and was thereafter able to travel successfully.

## 1. Case Presentation

A 68‐year‐old male was referred for recurrent syncope associated with air travel. He had a history of dyslipidemia, obstructive sleep apnea, nonalcoholic fatty liver disease, prostate cancer status postprostatectomy, and mild chronic obstructive pulmonary disease (COPD). His only medication was atorvastatin 20 mg daily. The patient was retired but previously worked as a construction manager. He had a 35‐pack‐year history of smoking but quit smoking 20 years prior. There was no family history of sudden cardiac death.

Over the last 12 years, he had been experiencing an increasing number of syncopal episodes while travelling by air, occurring one in every three flights. His last episode was 4 years prior during his last flight. During these episodes, he would start to feel significantly lightheaded (at times clammy and diaphoretic), and would then proceed to have a syncopal episode. He would have a quick recovery after loss of consciousness (LOC). He denied feeling claustrophobic during flying. He was feeling well before these episodes of air travel with no recent changes to his health. He does not have syncopal episodes outside of air travel. These syncopal episodes did not occur with movement of his neck or activities associated with pressure on the carotid sinus such as shaving. When he was a child, he did have a fear of needles and has had syncopal events associated with them, but that has since resolved.

**Figure 1:**
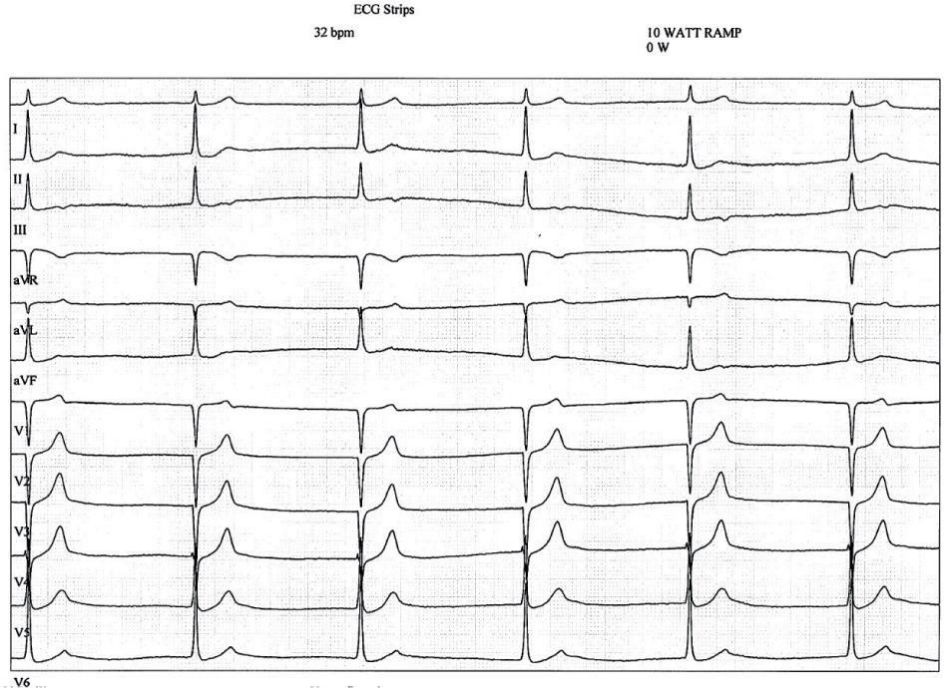
ECG during high altitude stimulation test at 15 min demonstrating junctional bradycardia with heart rate of 32 bpm with SpO2 of 89%. The patient developed brief presyncopal symptoms during this strip (lightheadedness and diaphoresis) which resolved after the application of oxygen supplementation.

The patient was asymptomatic and denied any cardiac symptoms. To investigate whether hypoxia triggered his episodes, respirology performed a high‐altitude stimulation test (HAST). From a baseline oxygen saturation (SpO2) of 96% and heart rate (HR) of 60 bpm, his SpO2 dropped to 86%–90% during testing. At 15 min, he developed presyncope (lightheadedness and diaphoresis) accompanied by an SpO2 of 89% and junctional bradycardia of 32 bpm (Figure 1). Syncope did not occur. Nasal oxygen administration rapidly resolved his symptoms, restoring his SpO2 to 97% and his HR to 62 bpm by 25 min.

**Figure 2:**
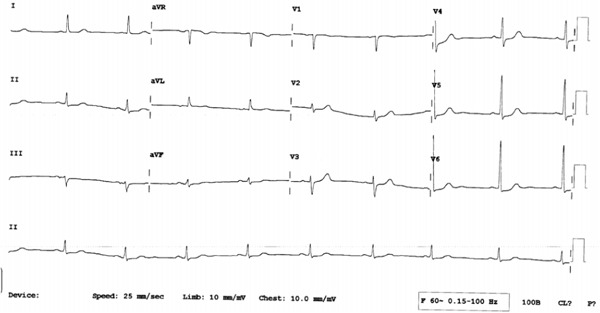
Baseline ECG demonstrating sinus bradycardia with heart rate of 55 bpm.

In clinic, his vital signs were as follows: blood pressure 146/78, HR 55 bpm, weight 73 kg, and BMI 24.3. He had an unremarkable cardiac and respiratory exam. Carotid sinus massage was performed and was negative. ECG in clinic revealed sinus bradycardia with HR of 55 bpm (Figure 2). Laboratory investigations revealed a normal CBC, electrolytes, renal function, and TSH. Echocardiogram revealed normal biventricular size and function with left ventricular ejection fraction (EF) of 55%. There were no significant valvular abnormalities and pulmonary artery systolic pressure of 22 mmHg. The patient underwent a standard Bruce protocol GXT, exercising for a total time of 8:59 min, achieving 10.1 METS. His maximum HR response was 155 bpm representing 101% of maximum predicted HR, and therefore, there was no evidence of chronotropic incompetence. There was no chest pain during the test. However, during peak exercise, there was diffuse ST depression, horizontal in nature of approximately 2 mm which prompted further testing. He subsequently underwent an exercise myocardial perfusion study which demonstrated normal myocardial perfusion with no evidence of myocardial ischemia.

Suspecting vasovagal syncope (VVS), a preventative management plan was devised to facilitate safe air travel. The patient was instructed to increase salt and fluid intake, perform counterpressure maneuvers, and take fludrocortisone 0.1 mg the night before his flights. To test this intervention, a repeat HAST was performed. The patient remained asymptomatic throughout the 20‐min test, maintaining a stable HR (64–69 bpm) and SpO2 (93%–95%). Following this successful trial, the patient has completed four symptom‐free flights utilizing these targeted pharmacological and nonpharmacological interventions.

## 2. Discussion

Syncope represents a sudden LOC characterized by the loss of postural tone and a swift recovery [1]. This is thought to be secondary to cerebral hypoperfusion, as termination of blood flow to the brain results in LOC within 10 s [1]. Reflex mediated‐syncope and orthostatic hypotension are the reason for at least one‐third of all syncopal episodes [1]. The types of reflex syncope primarily consist of situational triggers such as micturition or defecation related syncope, or VVS, for which common causes include prolonged standing, pain or emotional stress such as needles or the sight of blood [1]. These types of reflex syncope are postulated to occur from neural reflexes, either a vasodepressor or cardioinhibitory reflex (or a combination of the two) that cause inappropriate bradycardia and/or vasodilation, resulting in cerebral hypoperfusion and syncope [1].

In a patient with a structurally normal heart, syncope occurring during prolonged sitting or standing should raise high clinical suspicion for VVS as the primary diagnosis. Our diagnosis of VVS in this case is based on a prodrome consistent with VVS during flights, HAST that triggered this prodrome along with demonstration of a cardioinhibitory mechanism (bradycardia) during the HAST, and the absence of a clear alternative diagnosis after thorough cardiac investigation. A limitation of our clinical determination is the challenge in obtaining real‐time vital sign data on flights to confirm a vasovagal mechanism, including a vasodepressor or cardioinhibitory response that would result in hypotension and/or bradycardia in the moment. Another limitation of our clinical determination is the lack of tilt table testing. Other possibilities do include psychogenic pseudosyncope, which is hypothesized to be a presentation of conversion disorder, which is a physical manifestation of psychological stressors [2]. These patients present with the appearance of LOC without genuine LOC and are a diagnosis of exclusion [2]. Their prevalence in patients presenting for syncope evaluations has been reported to be up to 8% [2]. In the majority of cases, these patients have underlying psychiatric comorbidities. The possibility of this diagnosis was also considered in our patient, but he did not endorse any recent stressors in his life nor did he have a history of previous psychiatric conditions.

To our knowledge, our case is one of few reports of repeated air travel syncope, identifying air travel as a possible unique trigger of VVS in itself [3–7]. In a similar vein to our case, De and Davidson Ward described the case of a 6‐year‐old boy who experienced recurrent episodes of syncope during air travel and at altitude [7]. They postulated that several neurocardiogenic mechanisms may be at play due to the physiological adaptions that take place because of exposure to hypobaric hypoxia. The barometric pressure and partial pressure of oxygen decline with higher altitude in an aircraft cabin, simulating an FiO2 of approximately 15% [7, 8]. Hypoxia may therefore trigger increased ventilation, thereby causing hypocapnia [7]. This could result in reflex cerebral vasoconstriction and peripheral vasodilation [7, 9, 10]. Cerebral vasoconstriction may result in decreased cerebral oxygen delivery and inappropriate peripheral vasodilation may cause hypotension resulting in LOC [9, 10]. The above physiological cascade may also cause activation of the Bezold–Jarisch reflex, which is triggered by sensory receptors in the inferoposterior wall of the left ventricle [5]. When inappropriate vasodilation occurs, these sensory receptors sense decreased venous return to the heart, activating the Bezold–Jarisch reflex which enhances parasympathetic activity and causes bradycardia, resulting in LOC [5].

The HAST test in our case therefore stimulated the hypobaric hypoxia witnessed in aircraft cabins, and although healthy individuals can compensate for this decreased inspired oxygen, those with respiratory conditions such as COPD (as seen in our patient) are at risk for hypoxemia. Our patient′s HAST testing revealed mild hypoxemia with SpO2 of 86%–90% and slow junctional bradycardia, thereby provoking the patient′s symptoms, possibly through the above described mechanisms.

Behnoush et al. described a similar case of a patient who experienced five syncopal episodes all occurring during flight takeoff [6]. The patient was prescribed 5 mg of midodrine before departure and did not have any syncopal episodes during two subsequent flights. However, as opposed to the other cases reported in the literature where syncope was associated with higher altitude [3–5, 7], syncope in this case occurred during takeoff before the aircraft had reached higher altitude [6]. Therefore, the authors postulated that this mechanism may be slightly different than the hypobaric hypoxia witnessed in other cases, including our own case, and may be due to the gravitational force (G‐force) during takeoff [6]. Other postulated mechanisms for air travel syncope include postural hypotension, given that passengers are stationary for a prolonged period of time resulting in venous pooling in the lower extremities, with subsequent cerebral hypoperfusion when changing position rapidly [3]. However, this particular mechanism is less likely to be causative in our patient since his episodes occurred while he was seated, although not impossible. Claustrophobia is known to be a trigger for VVS as well, but our patient confirmed that he was not claustrophobic during air travel. Similarly, emotional distress is known to be a trigger for VVS, but our patient confirmed that he did not have a fear of flying.

Recognizing this trigger of VVS in our patient, we discussed both nonpharmacological measures and pharmacological options to mitigate his symptoms. As discussed in the Canadian Cardiovascular Society 2020 Clinical Practice Update, the 2018 European Society of Cardiology, and the 2017 American Heart Association guidelines, nonpharmacological measures consist of increased salt and water intake and counterpressure maneuvers of leg‐crossing, limb/abdominal contractions and squatting [11–13]. Avoiding known triggers of VVS in a particular patient is a recommendation, however given our patient′s unique trigger that was not possible [11]. Although compression garments are frequently recommended in clinical practice and in guidelines specifically to treat orthostatic hypotension, the recent COMFORTS‐II trial demonstrated that thigh‐high lower limb compression did not reduce the incidence of VVS recurrence [14]. Pharmacological options consist of fludrocortisone (0.2 mg daily) and midodrine (5–15 mg three times a day) [11]. Of note, given that hypoxia may have been a trigger for the vasovagal episode in our patient, oxygen therapy was considered. However, by the air travel guidelines for passengers with respiratory disease provided by the British Thoracic Society (BTS) in 2021, the patient would not qualify for oxygen therapy (in‐flight oxygen therapy only indicated when SpO2 < 85*%* on HAST) [8].

Given that our patient experienced VVS with a specific, predictable trigger, we chose to recommend that the fludrocortisone only be taken the night before his air travel, along with nonpharmacological methods. Fludrocortisone was chosen instead of midodrine because the patient desired a medication with a longer half‐life that would provide coverage for a full day of travel. Fludrocortisone may have mitigated syncope in our patient by interrupting the inappropriate physiological response during VVS [13, 15]. When inappropriate venodilation occurs causing decreased preload, the effect of fludrocortisone, by causing increased plasma volume, may counteract the physiological cascade that leads to hypotension and inappropriate bradycardia [13, 15].

Overall, this case demonstrates that air travel may represent a distinct situational trigger of VVS and may be mitigated by individualized preventative strategies including increased salt and water intake, counterpressure maneuvers, and the use of mineralocorticoids.

## Funding

No funding was received for this manuscript.

## Consent

The authors confirm that informed written consent was obtained for the submission and publication of this case report, including the accompanying text and images as per COPE guidelines.

## Conflicts of Interest

The authors declare no conflicts of interest.

## Data Availability

The data that support the findings of this study are available from the corresponding author upon reasonable request.
